# “NO FEAR, ALL FAITH”: RELIGIOUS COPING AND COVID-19 VACCINE HESITANCY AMONG OLDER AFRICAN AMERICANS

**DOI:** 10.1093/geroni/igad104.0509

**Published:** 2023-12-21

**Authors:** Antonius Skipper

**Affiliations:** Georgia State University, Atlanta, Georgia, United States

## Abstract

While COVID-19 had a devasting impact on many groups across the globe, older African Americans were especially affected by the disproportionate rates of morbidity and mortality associated with the virus. Faced with “double jeopardy” - the nature of being both old and Black - older African Americans were at an increased risk of COVID-19-related infection and death due to their age and factors associated with ethnicity (e.g., access to resources, distrust of healthcare). These risks led many health professionals to prioritize COVID-19 vaccine uptake for older African Americans. However, historically and contemporaneously, African Americans report some of the highest levels of vaccine hesitancy in comparison to other ethnic groups. Many older African Americans use religious coping as a source to alleviate the stress of health threats. Yet, the novelty of COVID-19 contributed to a limited understanding of how and why vaccine hesitant older African Americans used religious coping in response to the pandemic. The present study gathered qualitative data from 22 vaccine hesitant older African Americans to describe the role of religious coping in vaccine hesitancy. Data were analyzed using thematic analysis and inductive coding methods. Analyses identified several salient themes relative to religious coping and COVID-19 vaccine hesitancy, including (1) No Fear, All Faith, (2) Prayer Protects my Peace, and 3) My Faith Justifies my Hesitance. As vaccine uptake remains important for both older adults and African Americans, implications from this study highlight important considerations for navigating the nexus of religion and health with highly religious populations.

